# Anomalous Aortic Origin of Coronary Arteries from the Opposite Sinus: A Case Report

**DOI:** 10.7759/cureus.3092

**Published:** 2018-08-03

**Authors:** Nitin Sabharwal, Abhinav Saxena, Aleksandre Toreli, Vineet Meghrajani, Bilal Malik, Jacob Shani

**Affiliations:** 1 Internal Medicine, Maimonides Medical Center, Brooklyn, USA; 2 Department of Cardiology, Maimonides Medical Center, New York, USA; 3 Department of Cardiology, SUNY Downstate Medical Center, Brooklyn, USA; 4 Department of Internal Medicine, Maimonides Medical Center, Brooklyn, USA; 5 Department of Cardiology, Maimonides Medical Center, Brooklyn, USA

**Keywords:** anomalous coronary artery, right coronary cusp

## Abstract

Anomalous aortic origin of coronary arteries from the opposite sinus (AAOCA) is a rare finding which, when discovered, raises questions regarding its approach and management. Modern imaging techniques can help us to identify certain anatomical features of the anomalous coronary arteries to further classify them as benign or malignant anomalies. We present a case of a 64-year-old male who had an incidental finding of AAOCA with the left anterior descending artery arising from the right coronary cusp from an ostium anterior to the one that gave rise to both the left circumflex artery and right coronary artery (RCA). The patient was managed with a percutaneous coronary intervention for an obstructive disease of the RCA and was discharged with regular follow-ups.

## Introduction

Anomalous aortic origin of coronary arteries from the opposite sinus (AAOCA) is commonly considered in instances of sudden death in young individuals. In practice, the incidental finding of AAOCA leads to the following steps: imaging for the further study of the anatomy of the vessels, determining the clinical significance of the anomalous coronary artery, and making a decision around conservative or surgical management strategies [1]. The anatomical features that make an anomalous coronary artery benign or malignant and the mechanism by which they lead to compromised coronary flow have been discussed in this case report. 

## Case presentation

**Figure 1:**
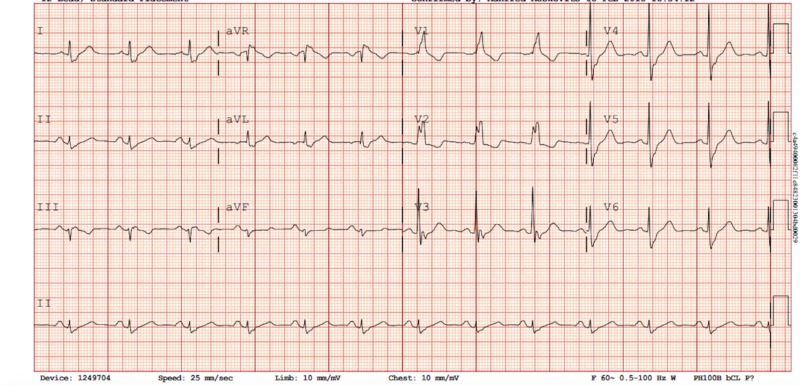
Electrocardiogram (ECG) on admission showing a sinus rhythm with a right bundle branch block and no ST segment or T wave changes indicative of ischemia.

**Figure 2:**
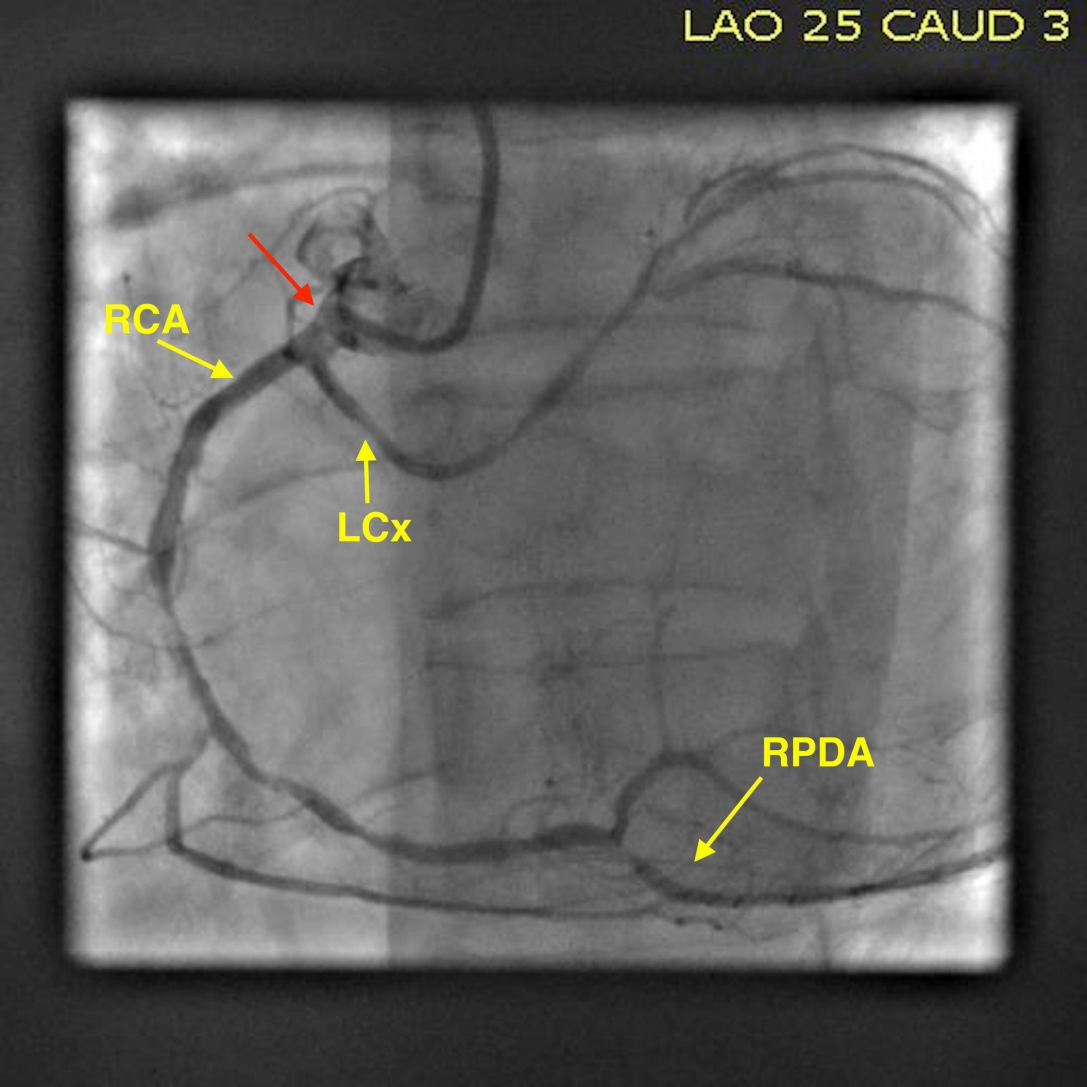
Coronary angiogram on admission Coronary angiogram findings demonstrating the diseased right coronary artery (RCA) and right posterior descending artery (RPDA). The red arrow points at the common origin of the left circumflex (LCx) and the right coronary artery (RCA).

**Figure 3:**
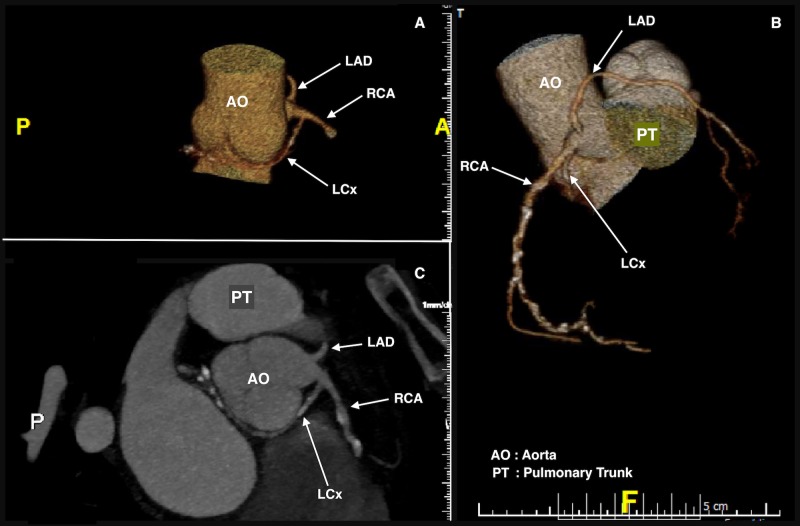
Cardiac Multi-detector Computed Tomography (MDCT) with 3-D reconstruction of the vessels. Panel A- demonstrates the course of the LCx as it swoops around the aorta after originating from a common trunk with the RCA. Panel B- demonstrates the origin and course of the Left Anterior Descending Artery ( LAD) anterior to the RCA  and over the pulmonary trunk. Panel C- computed tomography angiogram of the coronaries demonstrating the origin of the three vessels from the right coronary cusp and their relative locations to each other.

A 64-year-old male with a history of urothelial bladder cancer, who was a former smoker of a packet of cigarettes a day for 15 years, presented at his private medical doctor’s (PMD) practice with complaints of exertional chest pain. This chest pain was located in the middle of his chest, was pressure-like in nature, and was exacerbated with exertion and relieved with rest. The patient denied any associated diaphoresis or palpitations. An exercise stress test was performed, which showed inferior wall ischemic changes, and the patient was subsequently presented at our hospital for an elective angiogram. Upon admission, he was afebrile with a blood pressure of 147/85 mm/Hg and a regular heart rate of 79 beats/minute. The electrocardiogram (ECG) showed a sinus rhythm with a right bundle branch block and no ST segment or T wave changes indicative of ischemia (Figure 1). The lab analyses, such as troponin, lipid profiles, and fasting blood glucose, were within normal limits. The patient was referred to the catheterization lab for a coronary angiogram (CAG) through the right femoral artery. Cannulation of the right coronary ostia revealed the anomalous origin of the left circumflex artery (LCx) and the right coronary artery (RCA) from a shared ostium. The RCA was found to have 80% stenosis in the middle and distal segments with the right posterior descending artery (RPDA) having a 70% stenosis in the proximal segment (Figure 2). The LCx showed no significant stenosis. In the pursuit to locate the left coronary ostia, the discovery of the anomalous origin of the left anterior descending artery (LAD) from the right coronary cusp was made. It arose just anterior to the shared ostium of the RCA and LCx. The LAD had no stenosis. The procedure was thus aborted to perform a cardiac multidetector computed tomography (MDCT) to further study the anatomy of the patient in order to decide whether he would benefit from a surgical intervention or a percutaneous coronary intervention (PCI) of his obstructive coronary artery disease (CAD) (Figure 3). The MDCT confirmed the findings of the CAG. Considering the favorable anatomy of the LAD with no intramural course, a separate ostium, and no significant stenosis, the patient was referred back to the catheterization lab for a PCI. He received two drug-eluting stents in his RCA and one stent in his RPDA with excellent angiographic results. During the patient’s four-week follow-up appointment, he admitted to having better exercise tolerance and was free of exertional chest pain. 

## Discussion

The incidence of anomalous aortic origin of coronary arteries from the opposite sinus (AAOCA) is approximately 0.6% to 1.3% with the prevalence of the origin of the left coronary artery (LCA) from the right coronary cusp (RCC) reported to be about 0.03%. This varies depending upon the diagnostic criteria and reported population [2-5]. In fact, it can be considered to be more prevalent in the general population than reported since many of the individuals are asymptomatic and are never diagnosed. 

When an AAOCA is diagnosed, either with a transesophageal echocardiogram (TEE), computed tomography angiogram (CTA) or coronary angiogram, the next step is generally to further study the anatomy of the vessels. Non-invasive modern imaging techniques such as multidetector computed tomography (MDCT) and magnetic resonance imaging (MRI) allow us to clearly study the origin and route of the anomalous vessels with a three-dimensional reconstruction of the vessels (Figure 3). This equips the surgeons and interventional cardiologists with the information needed to determine the appropriate course of management [6-8]. 

Detailed imaging of the anatomy of the vessels not only helps in planning further management but also in identifying the anatomical features of an anomalous coronary artery (ACA) which make it benign or malignant. Features such as ostia type (separate, shared, or branched vessel), proximal vessel (normal, oval, or slit-like > 50% narrow), take-off angle (acute >45 degrees or non-acute <45 degrees), take-off level (above or below the aortic commissure), and length of narrowing of the ACA are taken into consideration [9]. AAOCA, be it in the RCA or the left coronary system, can take five common courses: interarterial, retroaortic, subpulmonic (septal) or prepulmonic, and retrocardiac. Amongst these, the interarterial course, which is when the ACA courses between the aorta and the pulmonary artery, is of higher clinical significance, mainly due to its association with angina and sudden death [10]. 

The higher prevalence of angina, arrhythmias, and sudden death occurring in patients with certain anatomical variants of AAOCA can be explained by a number of mechanisms [11]. Under the first mechanism, we take into account the acute take-off angle and slit-like ostium of origin. In simple terms, the ACA arising from the opposite sinus must take a sharp turn in order to be able to supply its respective side of the myocardium. As a result, the ostia may become slit-like and the take-off angle becomes acute. Alternatively, the acute take-off angle could lead to a slit-like ostium. Ultimately, this physiologically compromised anatomy can behave like an occlusion valve with the expansion of the aorta under exertion or arterial hypertension such that the patient may experience angina, arrhythmias and, in some cases, sudden death [12]. 

The second mechanism reported, which can co-exist with the first or vice versa, focuses on the pressure changes around the ACA, predominantly when taking an interarterial course. Interarterial courses have been further classified into: a) high interarterial or b) low interarterial, either of which may or may not have an intramural course. The high interarterial includes the ACA running between the aorta and pulmonary artery; the low interarterial is when the ACA runs between the aorta and the right ventricle outflow tract (RVOT). When the ACA runs between the aorta and the pulmonary artery, the expansion of the bigger arteries may compress the smaller coronary artery, the result of which is compromised coronary flow leading to angina, sudden death, and other major adverse cardiac events [10]. However, this explanation is questionable; the higher arterial pressure of the ACA, compared to the pulmonary trunk, could lead us to believe that the compression may only be a result of the aortic wall tension on the intramural segment of the ACA. Using the Laplace law, the radius of the aorta is much greater than the coronaries, leading to higher wall tension, which allows it to easily compress the intramural segment of the ACA in states of exertion or increased arterial hypertension leading to angina, arrhythmias or sudden death [11].

After gauging whether the ACA is benign or malignant in nature, a decision on whether to pursue surgical or conservative management, which includes medical treatment or percutaneous coronary intervention (PCI) in case of obstructive coronary artery disease (CAD), can be taken. Patients like the one above who presented with ischemia-like symptoms from obstructive coronary disease and favorable anatomy of the anomalous LAD arising from a separate ostium with no interarterial or intramural course may be managed non-surgically with a PCI [13]. 

## Conclusions

In conclusion, the incidental finding of AAOCA remains a matter of discussion as there is no clear approach or management strategy. In cases of an obstructive CAD leading to angina, as in the reported case, weighing the ostia, take-off angle, and the course of the ACA while making an informed decision regarding whether to perform surgery or PCI is advised. 
